# Isolation of a reassortant H13N2 virus from a mallard fecal sample in South Korea

**DOI:** 10.1186/1743-422X-9-133

**Published:** 2012-07-23

**Authors:** Hyun-Mi Kang, Jun-Gu Choi, Min-Chul Kim, Hye-Ryoung Kim, Jae-Ku Oem, You-Chan Bae, Mi-Ra Paek, Jun-Hun Kwon, Youn-Jeong Lee

**Affiliations:** 1 Avian Disease Division, Animal, Plant and Fisheries Quarantine and Inspection Agency, 175 Anyangro, Anyangsi, Gyeonggido, 430-757, Republic of Korea; 2 Animal Disease Diagnostic Division, Animal, Plant and Fisheries Quarantine and Inspection Agency, 175 Anyangro, Anyangsi, Gyeonggido, 430-757, Republic of Korea

**Keywords:** Avian influenza virus, H13N2, Mallard, Phylogenetic analysis, South Korea

## Abstract

**Background:**

Virus subtype H13N2, A/mallard/Kr/SH38-45/2010 (H13N2), was first isolated from a mallard fecal sample in South Korea.

**Results:**

Phylogenetic analysis of all eight viral genes revealed that this virus emerged by genetic mixing between Eurasian and North American gene pools, and possibly between wild ducks and gulls. The H13 and N2 surface genes clustered together in a group with Eurasian isolates from gulls and wild birds, respectively. The PB2, PA, NP, M and NS segments belonged to the Eurasian lineage, whereas the PB1 gene clustered in the North American lineage. Furthermore, they showed a bird-dependent pattern in phylogenetic analysis: the M gene was similar to subtype H13 viruses within gulls, whereas other segments were similar to avian influenza viruses of other subtypes from wild ducks.

**Conclusions:**

The data suggests that the novel reassortant H13N2 virus isolated in South Korea might have emerged by genetic reassortment between intercontinental and interspecies transmission in wild birds.

## Findings

Wild birds in the orders *Anseriformes* (ducks, geese and swans) and *Charadriiformes* (gulls, terns and shorebirds) collectively are the natural reservoir for all known subtypes of avian influenza viruses [1,2]. Influenza virus subtype H13 seems to be highly gull-associated [2,3] and is rarely found in other avian species that are natural hosts of influenza A virus, such as ducks and geese [4,5]. Since the first report from the United States in 1982, H13 influenza virus has been detected in Eurasia, and North and South America, with corresponding evolutionary lineages [5].

In South Korea, fresh fecal samples from migratory wild birds were collected during wintering seasons, from September 2010 to the following March to survey for avian influenza viruses (AIVs) in four wetlands (Miho-cheon, Pungse-cheon, Shihwa-ho and Cheonsu-man). Fresh fecal samples from migratory birds such as mallard (*Anas platyrhynchos*) and spot-billed duck (*Anas poecilorhyncha*), visually identified, were collected from the environment. However, as we actually collected samples from a group of birds after observing defecation, we need confirming bird species by mitochondrial analysis. AIVs were isolated by embryonic egg inoculation with fecal samples obtained from migratory wild birds and the presence of AIV was determined using a hemagglutination assay after incubating the eggs at 37 °C for 4–5 days. AIVs were subtyped using reverse transcription-polymerase chain reaction and DNA sequencing. A barcoding system utilizing mitochondrial DNA of bird feces [6,7] was employed to determine the host species. The viral genes were sequenced and analyzed as previously described [8]. The gene sequences of this H13 virus have been deposited in GenBank under accession no. (JX030402 ~ JX030409).

Within this collection, a total of 2,227 fecal samples were collected and AIV was recovered from 59 samples. H13N2 virus [A/mallard/Kr/SH38-45/2010 (H13N2), designated Md/SH38-45 (H13N2)] was identified. Although intensive surveillance on wild birds for avian influenza has been performed, there has been no prior report of the isolation of H13 virus in South Korea [9-11]. Subtypes H13 and H16 may be highly associated with gulls [3]. Therefore, the failure to detect subtype H13 may be associated with the AIV surveillance program in South Korea, which has largely focused on fecal samples of migratory wild ducks. To our knowledge, this is the first report of the isolation of a H13 subtype virus in South Korea.

**Figure 1:**
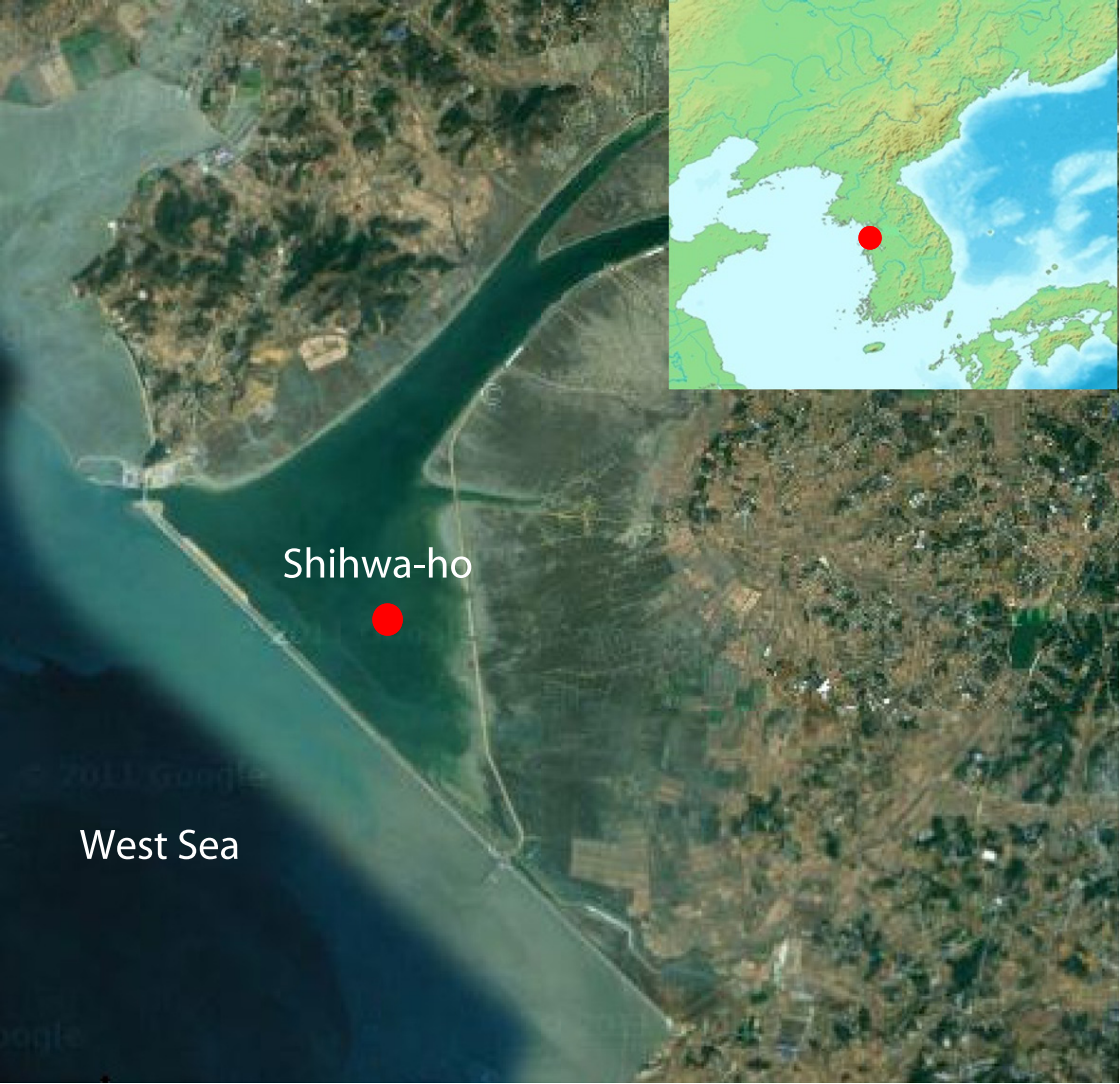
**Location of sampling site of Md/SH38-45 (H13N2) virus in South Korea (inset map).** The wetland site, Shihwa-ho, is a reclaimed lake adjacent to the West Sea in South Korea (larger map).

South Korea is important as a wintering locale for wild birds in the order *Anseriformes* such as ducks and geese, and as a resting point for *Charadriiformes* during their migration to Australia [9,12]. One of the wetlands from which the novel H13 virus was isolated is Shihwa-ho, a reclaimed lake adjacent to the West Sea in South Korea (Figure 1). Shihwa-ho is a shared habitat between the *Anseriformes* such as ducks and geese, and shorebirds such as gulls. Thus, the wetland may be an important location for interspecies transmission, further increasing the opportunity for transmission and reassortment of avian influenza viruses.

**Figure 2:**
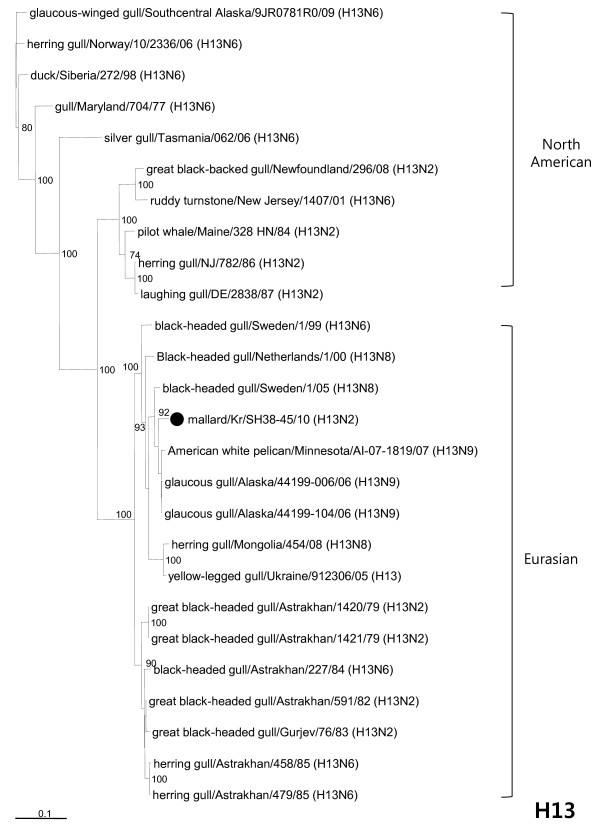
**Phylogenetic tree of the H13 HA gene.** Placement of the Md/SH38-45 (H13N2) virus isolate is indicated by a filled circle. The nucleotide sequences were analyzed using Clustal X (version 1.83) and phylogenetic trees were constructed by the neighbor-joining method using the VectorNTI Advance program (Invitrogen, Carlsbad, CA). The robustness of groupings was assessed by bootstrap resampling of 1000 replicate trees.

The phylogenetic identification of Md/SH38-45 (H13N2) virus from a mallard fecal sample indicates that intercontinental (Eurasia and America) and interspecies (gull and wild duck) reassortment events (or transmission) have occurred (Table 1). As shown in Figure 2, subytpe H13 viruses available on GenBank were mainly isolated from gulls and rarely from wild ducks or other animals in both the Eurasian and North American lineage in the phylogenetic tree. Md/SH38-45 (H13N2) virus was isolated from mallard (wild duck) and clustered together in a group with Eurasian isolates from gulls (Figure 2), which showed a cleavage site consistent with a low pathogenic AI (VPAISNR/GLF) at the analysis of the deduced HA protein sequence. The NA gene grouped with the Eurasian lineage and exhibited close genetic relationships to AIVs from wild birds, whereas they were distinguishable from the H9N2 isolates in poultry ( Additional file 1: Figure S1a).

**Table 1:** Lineage analysis of Md/SH38-45 (H13N2) virus

**Genes**		**Lineage**		**Species**	
		**Eurasian**	**North American**	**Wild duck**	**Gull**
Surface genes	HA	O			O
	NA	O		O	
Internal genes	PB2	O		O	
	PB1		O	O	
	PA	O		O	
	NP	O		O	
	M	O			O
	NS	O		O	

The present analyses revealed that internal genes, polymerase subunit PB2 (PB2), polymerase acidic (PA), nucleoprotein (NP) and nonstructural protein (NS) genes belonged to the Eurasian lineage, and that they were similar to AIVs from wild ducks ( Additional file 1: Figures S1b, d, e, g). The M segment clustered in a group with the Eurasian lineage and was similar to subtype H13 viruses within gulls ( Additional file 1: Figure S1f). In contrast, the PB1 gene clustered in the North American lineage, unlike the other genes, and showed high sequence similarity with the isolates from wild ducks ( Additional file 1: Figure S1c). In addition, two viruses [glaucous gull/Alaska/44199-006/06 (H13N9) and glaucous gull/Alaska/44199-104/06 (H13N9)] from Alaska [13] that were included in the phylogenetic analyses were closely related with four genes [HA (97.1–97.2%), NP (98.1–98.3%), M (96.6–96.7%), and NS (98.0–98.1%) of Md/SH38-45 (H13N2) virus, showing high sequence similarity.

Together, our findings indicate that the overlap of the East Asian/Australian and the Pacific Americas migratory flyways may serve as not only a link between continental AIV gene pools in waterfowl hosts, but also in gulls [7,13]. The PA segment of internal genes belonged to the same lineage (94.8–96.6% homology) with the Gs/GD-like lineage of H5N1 highly pathogenic avian influenza viruses ( Additional file 1: Figure S1d). This result is consistent with an earlier report that the multiple gene pools of wild birds provide Gs/GD-like viruses with their genes in part by reassortment events [14].

In conclusion, it seems likely that reassortment events of Md/SH38-45 (H13N2) virus have emerged as the result of genetic mixing between Eurasian and North American gene pools, and between wild ducks and gulls.

## Competing interests

The authors declare that they have no competing interests.

## Authors’ contributions

KHM conducted sample collection, virus isolation, virus identification, genome sequencing, phylogenetic analysis and drafted the manuscript. CJG, KMC, KHR, OJK, BYC and PMR participated in sample collection and virus isolation. KJH helped design the wild bird surveillance. LYJ designed the wild bird surveillance, conducted data analysis and provided final approval of the manuscript. All authors read and approved the final manuscript.

## Supplementary Material

Additional file 1 **Figure S1. Phylogenetic trees of a (N2), b (PB2), c (PB1), d (PA), e (NP), f (M) and g (NS).** Md/SH38-45 (H13N2) virus is indicated by a filled circled. The nucleotide sequences were analyzed using Clustal X (version 1.83) and phylogenetic trees were constructed by the neighbor-joining method. The robustness of groupings was assessed by bootstrap resampling of 1000 replicate trees. Click here for file
