# Supplementary material for: Isolation of a reassortant H13N2 virus from a mallard fecal sample in South Korea
Source: Virol J. 2012 Jul 23;9:133. doi: 10.1186/1743-422X-9-133 (PMC3423068; doi:10.1186/1743-422X-9-133)
Supplement: Additional file 1 — Figure S1. Phylogenetic trees of a (N2), b (PB2), c (PB1), d (PA), e (NP), f (M) and g (NS). Md/SH38-45 (H13N2) virus is indicated by a filled circled. The nucleotide sequences were analyzed using Clustal X (version 1.83) and phylogenetic trees were constructed by the neighbor-joining method. The robustness of groupings was assessed by bootstrap resampling of 1000 replicate trees. [file 1743-422X-9-133-S1.ppt]

## Slide 1
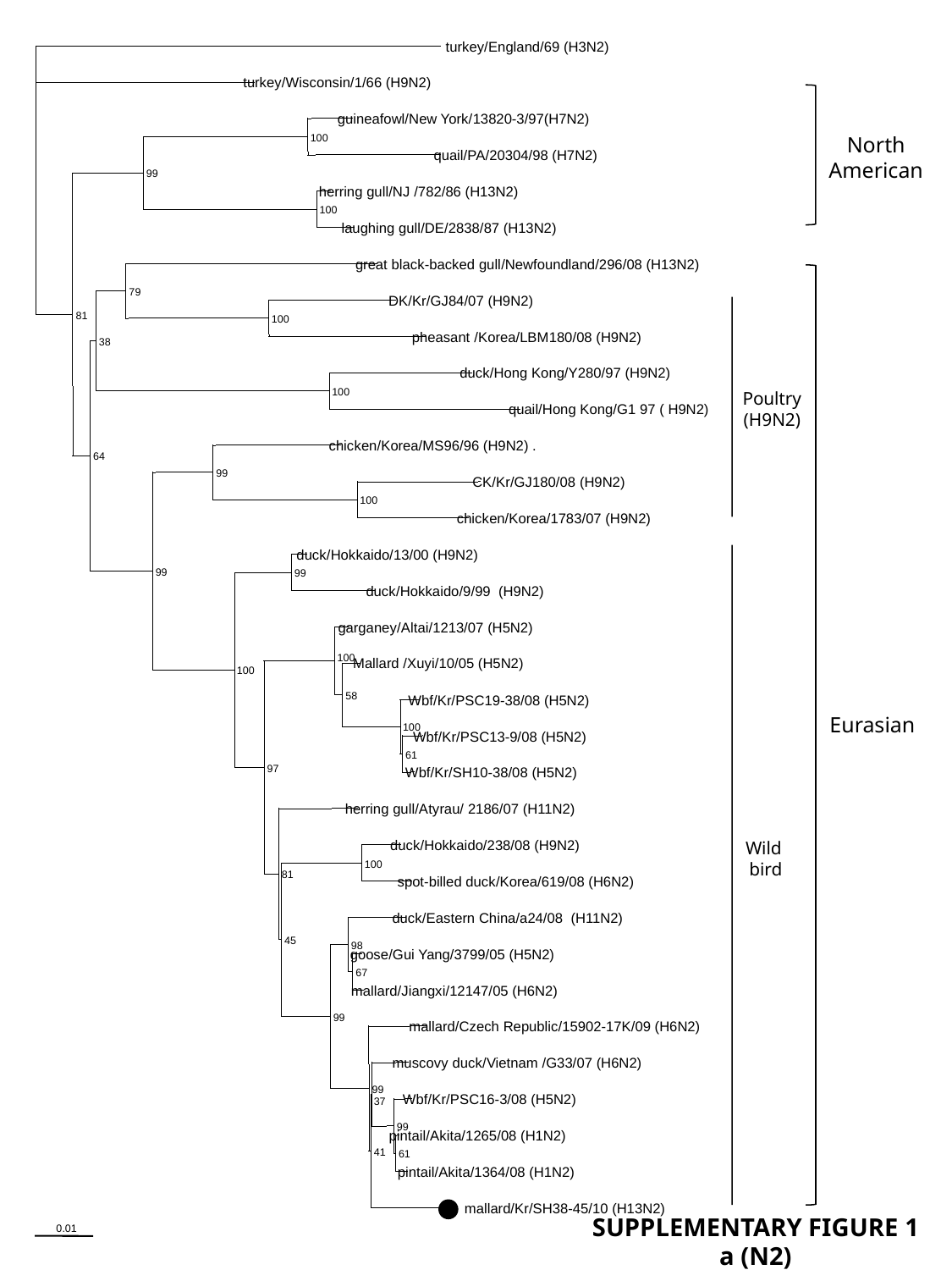

turkey/England/69 (H3N2)
turkey/Wisconsin/1/66 (H9N2)
guineafowl/New York/13820-3/97(H7N2)
100
quail/PA/20304/98 (H7N2)
99
herring gull/NJ /782/86 (H13N2)
100
laughing gull/DE/2838/87 (H13N2)
great black-backed gull/Newfoundland/296/08 (H13N2)
79
DK/Kr/GJ84/07 (H9N2)
81
100
pheasant /Korea/LBM180/08 (H9N2)
38
duck/Hong Kong/Y280/97 (H9N2)
100
quail/Hong Kong/G1 97 ( H9N2)
chicken/Korea/MS96/96 (H9N2) .
64
99
CK/Kr/GJ180/08 (H9N2)
100
chicken/Korea/1783/07 (H9N2)
duck/Hokkaido/13/00 (H9N2)
99
99
duck/Hokkaido/9/99 (H9N2)
garganey/Altai/1213/07 (H5N2)
100
Mallard /Xuyi/10/05 (H5N2)
100
58
Wbf/Kr/PSC19-38/08 (H5N2)
100
Wbf/Kr/PSC13-9/08 (H5N2)
61
97
Wbf/Kr/SH10-38/08 (H5N2)
herring gull/Atyrau/ 2186/07 (H11N2)
duck/Hokkaido/238/08 (H9N2)
100
81
spot-billed duck/Korea/619/08 (H6N2)
duck/Eastern China/a24/08 (H11N2)
45
98
goose/Gui Yang/3799/05 (H5N2)
67
mallard/Jiangxi/12147/05 (H6N2)
99
mallard/Czech Republic/15902-17K/09 (H6N2)
muscovy duck/Vietnam /G33/07 (H6N2)
99
Wbf/Kr/PSC16-3/08 (H5N2)
37
99
pintail/Akita/1265/08 (H1N2)
41
61
pintail/Akita/1364/08 (H1N2)
 mallard/Kr/SH38-45/10 (H13N2)
SUPPLEMENTARY FIGURE 1
a (N2)
0.01
North
American
Poultry
(H9N2)
Eurasian
Wild
bird

## Slide 2
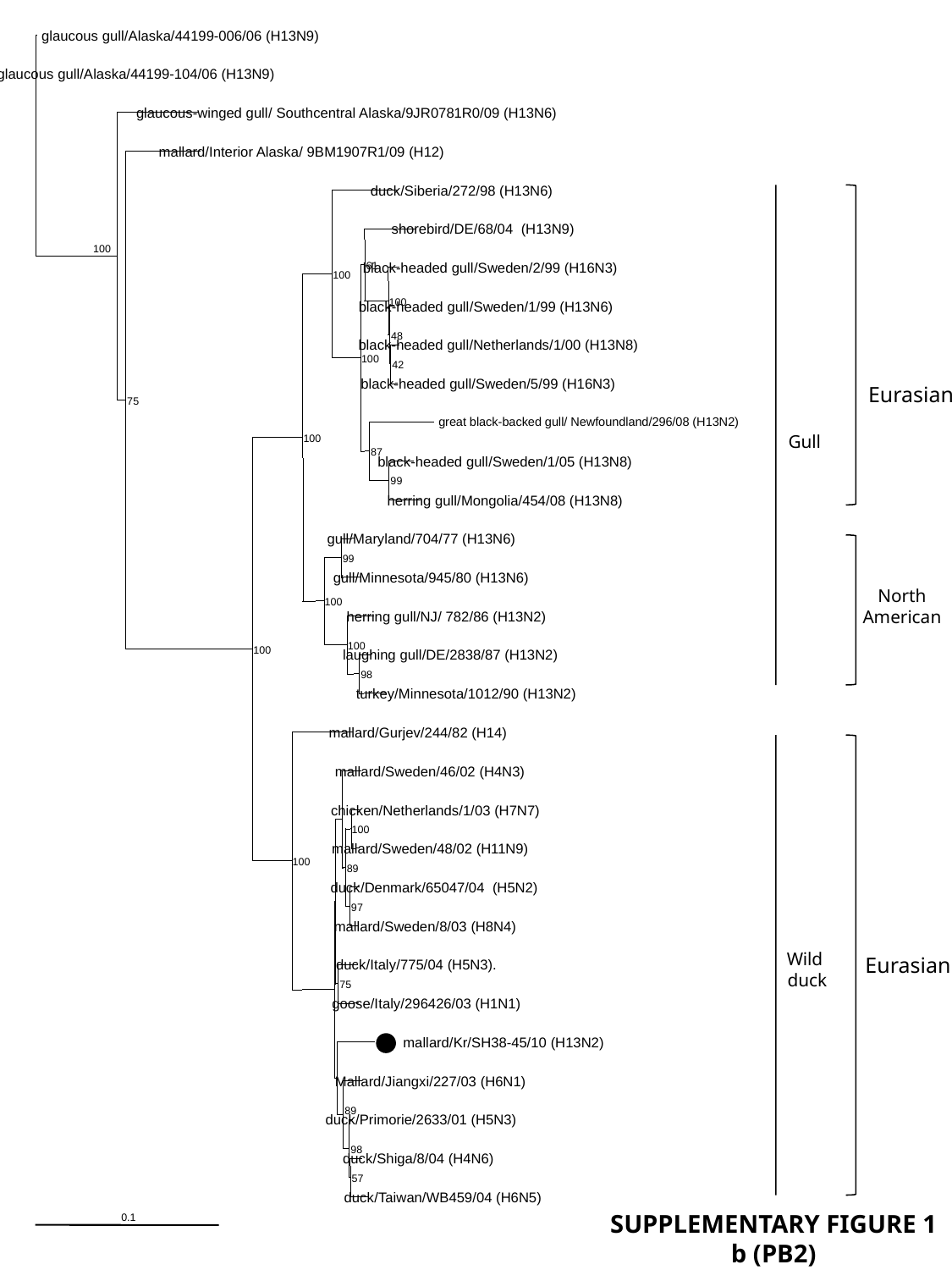

glaucous gull/Alaska/44199-006/06 (H13N9)
glaucous gull/Alaska/44199-104/06 (H13N9)
glaucous-winged gull/ Southcentral Alaska/9JR0781R0/09 (H13N6)
mallard/Interior Alaska/ 9BM1907R1/09 (H12)
duck/Siberia/272/98 (H13N6)
shorebird/DE/68/04 (H13N9)
100
black-headed gull/Sweden/2/99 (H16N3)
61
100
100
black-headed gull/Sweden/1/99 (H13N6)
48
black-headed gull/Netherlands/1/00 (H13N8)
100
42
black-headed gull/Sweden/5/99 (H16N3)
75
great black-backed gull/ Newfoundland/296/08 (H13N2)
100
87
black-headed gull/Sweden/1/05 (H13N8)
99
herring gull/Mongolia/454/08 (H13N8)
gull/Maryland/704/77 (H13N6)
99
gull/Minnesota/945/80 (H13N6)
100
herring gull/NJ/ 782/86 (H13N2)
100
100
laughing gull/DE/2838/87 (H13N2)
98
turkey/Minnesota/1012/90 (H13N2)
mallard/Gurjev/244/82 (H14)
mallard/Sweden/46/02 (H4N3)
chicken/Netherlands/1/03 (H7N7)
100
mallard/Sweden/48/02 (H11N9)
100
89
duck/Denmark/65047/04 (H5N2)
97
mallard/Sweden/8/03 (H8N4)
duck/Italy/775/04 (H5N3).
75
goose/Italy/296426/03 (H1N1)
 mallard/Kr/SH38-45/10 (H13N2)
Mallard/Jiangxi/227/03 (H6N1)
89
duck/Primorie/2633/01 (H5N3)
98
duck/Shiga/8/04 (H4N6)
57
duck/Taiwan/WB459/04 (H6N5)
0.1
Eurasian
Gull
Wild
duck
Eurasian
SUPPLEMENTARY FIGURE 1
b (PB2)
North
American

## Slide 3
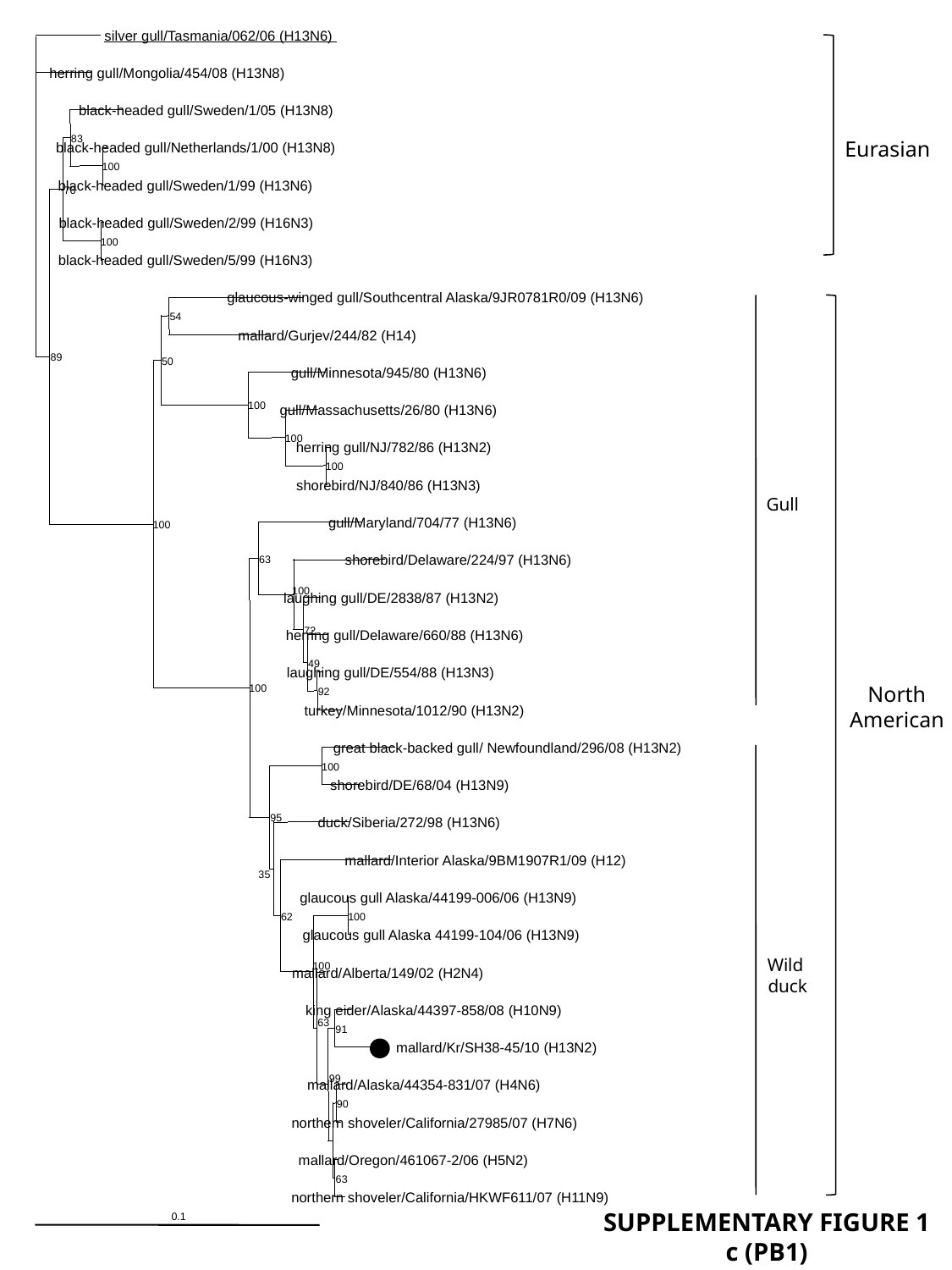

silver gull/Tasmania/062/06 (H13N6)
herring gull/Mongolia/454/08 (H13N8)
black-headed gull/Sweden/1/05 (H13N8)
83
black-headed gull/Netherlands/1/00 (H13N8)
100
black-headed gull/Sweden/1/99 (H13N6)
76
black-headed gull/Sweden/2/99 (H16N3)
100
black-headed gull/Sweden/5/99 (H16N3)
glaucous-winged gull/Southcentral Alaska/9JR0781R0/09 (H13N6)
54
mallard/Gurjev/244/82 (H14)
89
50
gull/Minnesota/945/80 (H13N6)
100
gull/Massachusetts/26/80 (H13N6)
100
herring gull/NJ/782/86 (H13N2)
100
shorebird/NJ/840/86 (H13N3)
gull/Maryland/704/77 (H13N6)
100
shorebird/Delaware/224/97 (H13N6)
63
100
laughing gull/DE/2838/87 (H13N2)
72
herring gull/Delaware/660/88 (H13N6)
49
laughing gull/DE/554/88 (H13N3)
100
92
turkey/Minnesota/1012/90 (H13N2)
great black-backed gull/ Newfoundland/296/08 (H13N2)
100
shorebird/DE/68/04 (H13N9)
95
duck/Siberia/272/98 (H13N6)
mallard/Interior Alaska/9BM1907R1/09 (H12)
35
glaucous gull Alaska/44199-006/06 (H13N9)
62
100
glaucous gull Alaska 44199-104/06 (H13N9)
100
mallard/Alberta/149/02 (H2N4)
king eider/Alaska/44397-858/08 (H10N9)
63
91
 mallard/Kr/SH38-45/10 (H13N2)
99
mallard/Alaska/44354-831/07 (H4N6)
90
northern shoveler/California/27985/07 (H7N6)
mallard/Oregon/461067-2/06 (H5N2)
63
northern shoveler/California/HKWF611/07 (H11N9)
0.1
Eurasian
Gull
North
American
Wild
 duck
SUPPLEMENTARY FIGURE 1
c (PB1)

## Slide 4
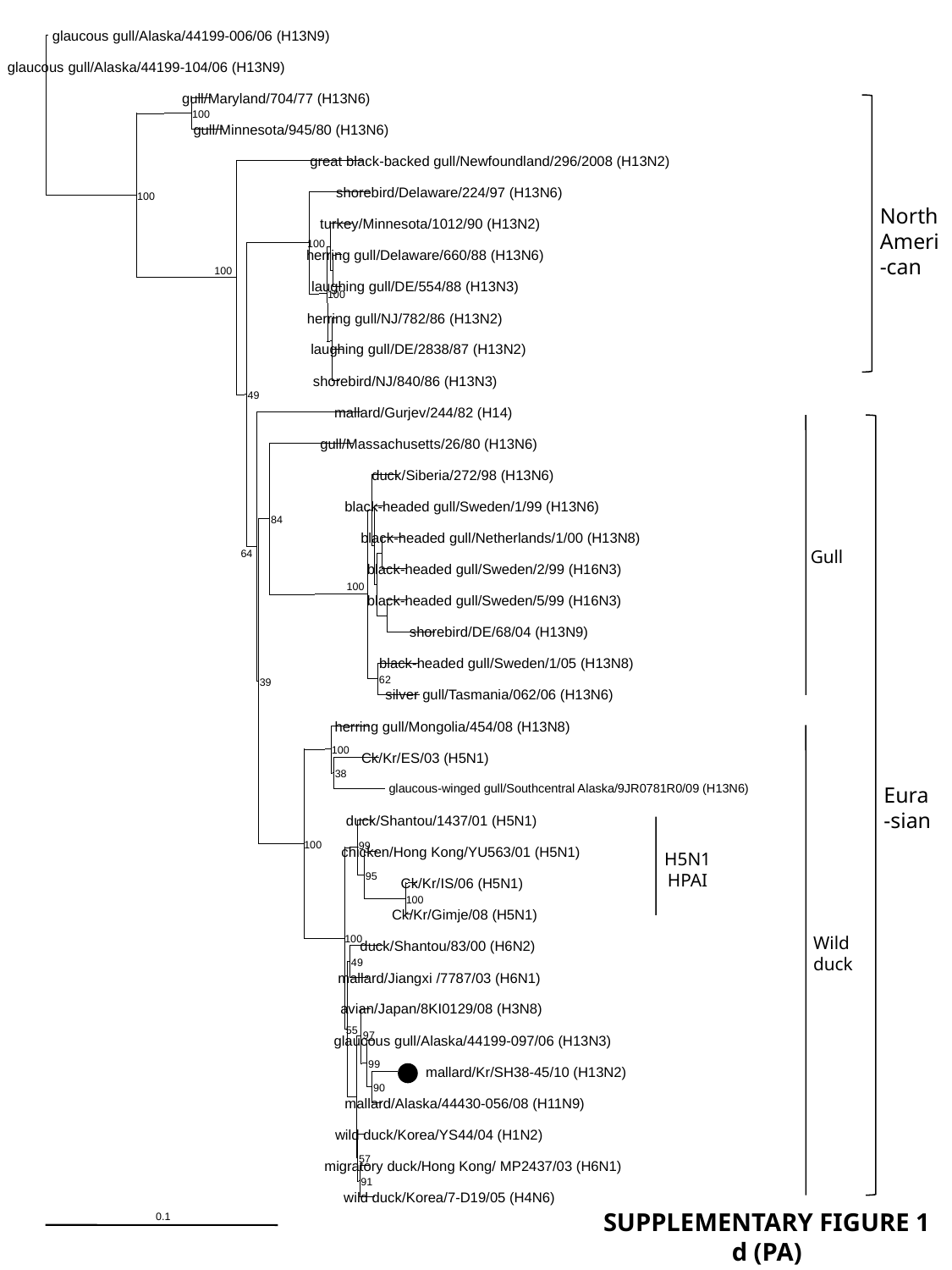

glaucous gull/Alaska/44199-006/06 (H13N9)
glaucous gull/Alaska/44199-104/06 (H13N9)
gull/Maryland/704/77 (H13N6)
100
gull/Minnesota/945/80 (H13N6)
great black-backed gull/Newfoundland/296/2008 (H13N2)
shorebird/Delaware/224/97 (H13N6)
100
turkey/Minnesota/1012/90 (H13N2)
100
herring gull/Delaware/660/88 (H13N6)
100
laughing gull/DE/554/88 (H13N3)
100
herring gull/NJ/782/86 (H13N2)
laughing gull/DE/2838/87 (H13N2)
shorebird/NJ/840/86 (H13N3)
49
mallard/Gurjev/244/82 (H14)
gull/Massachusetts/26/80 (H13N6)
duck/Siberia/272/98 (H13N6)
black-headed gull/Sweden/1/99 (H13N6)
84
black-headed gull/Netherlands/1/00 (H13N8)
64
black-headed gull/Sweden/2/99 (H16N3)
100
black-headed gull/Sweden/5/99 (H16N3)
shorebird/DE/68/04 (H13N9)
black-headed gull/Sweden/1/05 (H13N8)
62
39
silver gull/Tasmania/062/06 (H13N6)
herring gull/Mongolia/454/08 (H13N8)
100
Ck/Kr/ES/03 (H5N1)
38
glaucous-winged gull/Southcentral Alaska/9JR0781R0/09 (H13N6)
duck/Shantou/1437/01 (H5N1)
100
99
chicken/Hong Kong/YU563/01 (H5N1)
95
Ck/Kr/IS/06 (H5N1)
100
Ck/Kr/Gimje/08 (H5N1)
100
duck/Shantou/83/00 (H6N2)
49
mallard/Jiangxi /7787/03 (H6N1)
avian/Japan/8KI0129/08 (H3N8)
55
97
glaucous gull/Alaska/44199-097/06 (H13N3)
99
 mallard/Kr/SH38-45/10 (H13N2)
90
mallard/Alaska/44430-056/08 (H11N9)
wild duck/Korea/YS44/04 (H1N2)
57
migratory duck/Hong Kong/ MP2437/03 (H6N1)
91
wild duck/Korea/7-D19/05 (H4N6)
0.1
North
Ameri
-can
Gull
Eura
-sian
H5N1
HPAI
Wild
duck
SUPPLEMENTARY FIGURE 1
d (PA)

## Slide 5
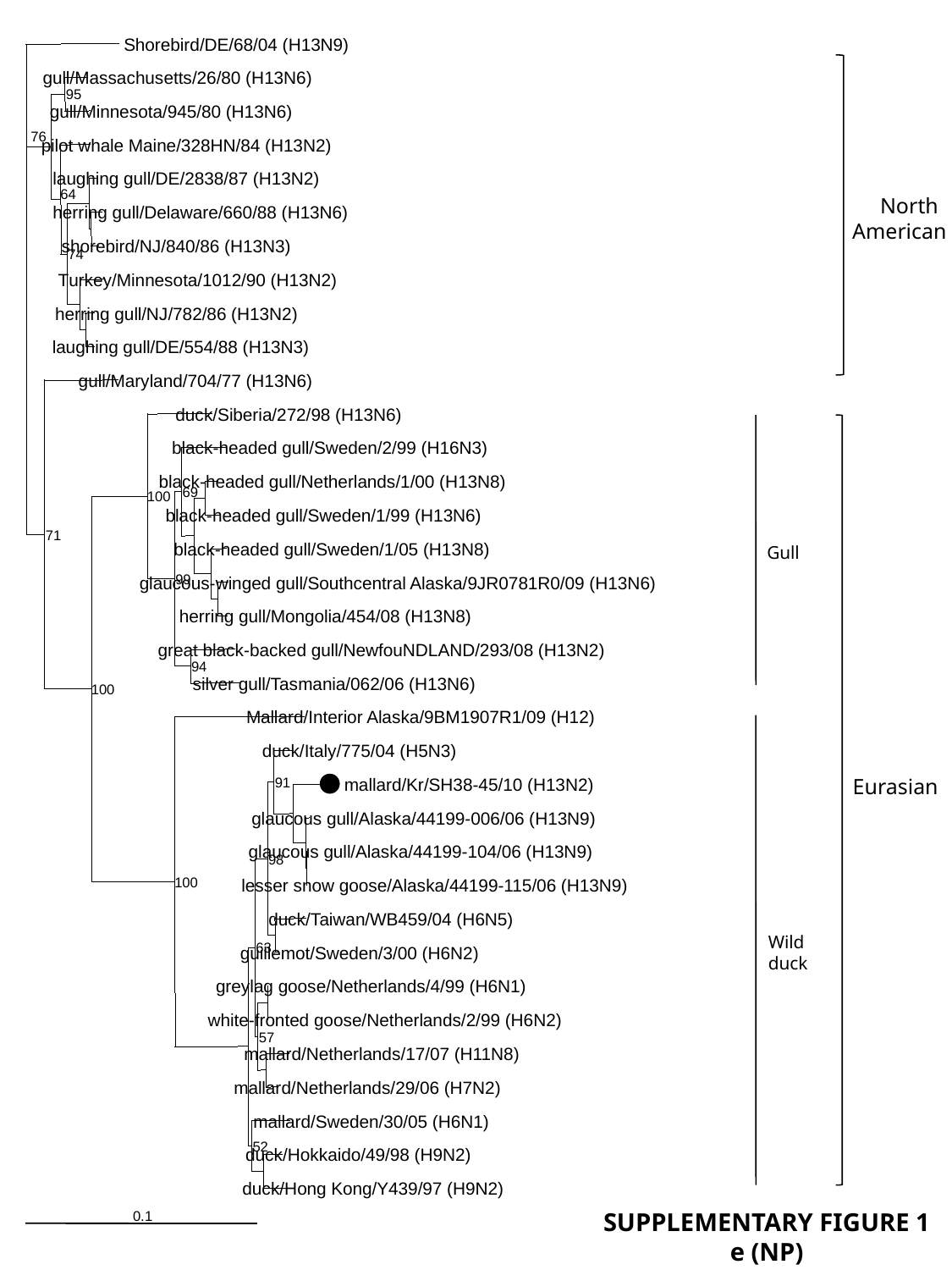

Shorebird/DE/68/04 (H13N9)
gull/Massachusetts/26/80 (H13N6)
95
gull/Minnesota/945/80 (H13N6)
76
pilot whale Maine/328HN/84 (H13N2)
laughing gull/DE/2838/87 (H13N2)
64
North
American
herring gull/Delaware/660/88 (H13N6)
shorebird/NJ/840/86 (H13N3)
74
Turkey/Minnesota/1012/90 (H13N2)
herring gull/NJ/782/86 (H13N2)
laughing gull/DE/554/88 (H13N3)
gull/Maryland/704/77 (H13N6)
duck/Siberia/272/98 (H13N6)
black-headed gull/Sweden/2/99 (H16N3)
black-headed gull/Netherlands/1/00 (H13N8)
69
100
black-headed gull/Sweden/1/99 (H13N6)
71
Gull
black-headed gull/Sweden/1/05 (H13N8)
99
glaucous-winged gull/Southcentral Alaska/9JR0781R0/09 (H13N6)
herring gull/Mongolia/454/08 (H13N8)
great black-backed gull/NewfouNDLAND/293/08 (H13N2)
94
silver gull/Tasmania/062/06 (H13N6)
100
Mallard/Interior Alaska/9BM1907R1/09 (H12)
duck/Italy/775/04 (H5N3)
Eurasian
 mallard/Kr/SH38-45/10 (H13N2)
91
glaucous gull/Alaska/44199-006/06 (H13N9)
glaucous gull/Alaska/44199-104/06 (H13N9)
98
100
lesser snow goose/Alaska/44199-115/06 (H13N9)
duck/Taiwan/WB459/04 (H6N5)
Wild
duck
63
guillemot/Sweden/3/00 (H6N2)
greylag goose/Netherlands/4/99 (H6N1)
white-fronted goose/Netherlands/2/99 (H6N2)
57
mallard/Netherlands/17/07 (H11N8)
mallard/Netherlands/29/06 (H7N2)
mallard/Sweden/30/05 (H6N1)
52
duck/Hokkaido/49/98 (H9N2)
duck/Hong Kong/Y439/97 (H9N2)
SUPPLEMENTARY FIGURE 1
e (NP)
0.1

## Slide 6
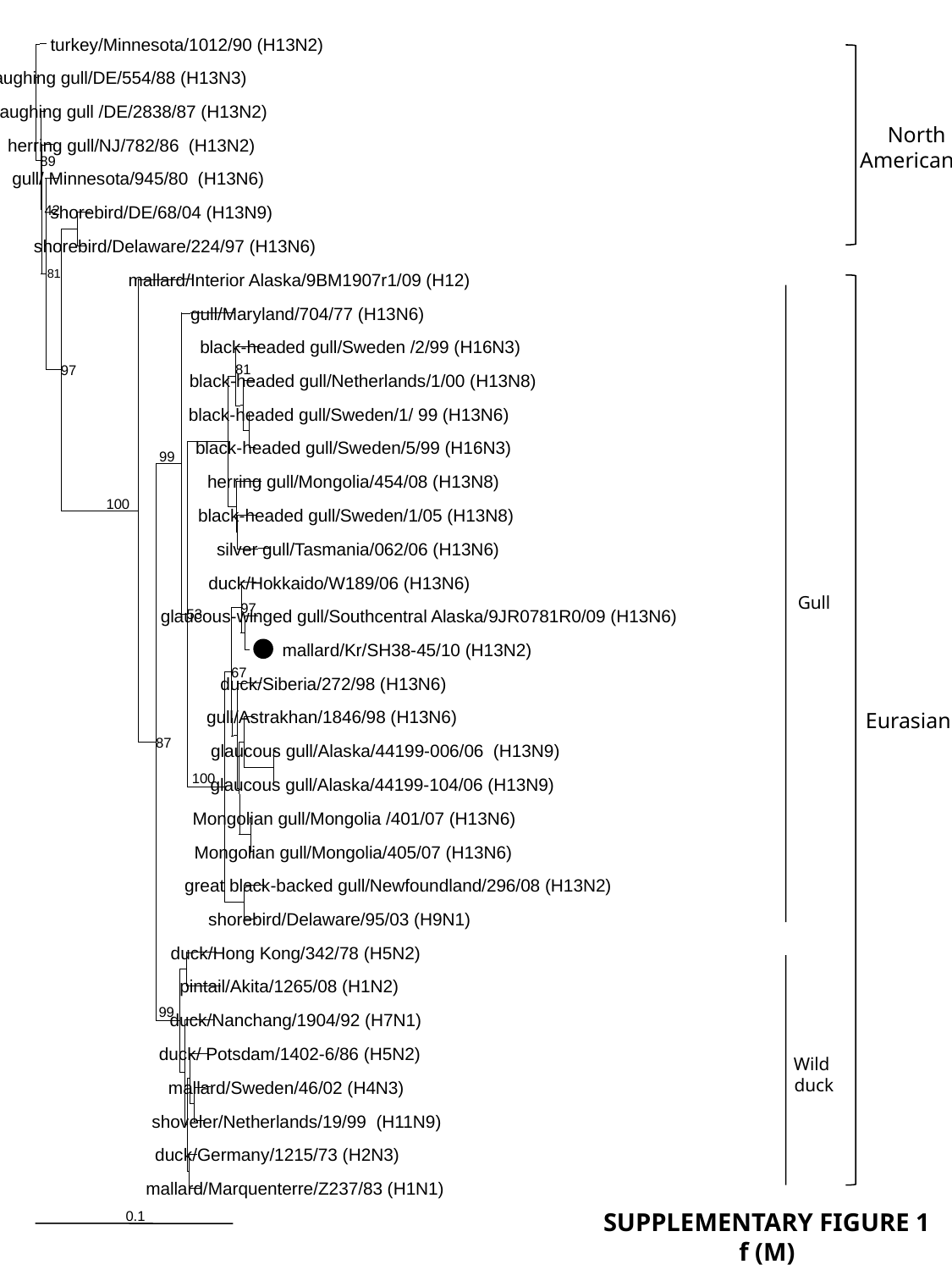

turkey/Minnesota/1012/90 (H13N2)
laughing gull/DE/554/88 (H13N3)
laughing gull /DE/2838/87 (H13N2)
herring gull/NJ/782/86 (H13N2)
89
gull/ Minnesota/945/80 (H13N6)
42
shorebird/DE/68/04 (H13N9)
shorebird/Delaware/224/97 (H13N6)
81
mallard/Interior Alaska/9BM1907r1/09 (H12)
gull/Maryland/704/77 (H13N6)
black-headed gull/Sweden /2/99 (H16N3)
81
97
black-headed gull/Netherlands/1/00 (H13N8)
black-headed gull/Sweden/1/ 99 (H13N6)
black-headed gull/Sweden/5/99 (H16N3)
99
herring gull/Mongolia/454/08 (H13N8)
100
black-headed gull/Sweden/1/05 (H13N8)
silver gull/Tasmania/062/06 (H13N6)
duck/Hokkaido/W189/06 (H13N6)
97
glaucous-winged gull/Southcentral Alaska/9JR0781R0/09 (H13N6)
53
 mallard/Kr/SH38-45/10 (H13N2)
67
duck/Siberia/272/98 (H13N6)
gull/Astrakhan/1846/98 (H13N6)
87
glaucous gull/Alaska/44199-006/06 (H13N9)
100
glaucous gull/Alaska/44199-104/06 (H13N9)
Mongolian gull/Mongolia /401/07 (H13N6)
Mongolian gull/Mongolia/405/07 (H13N6)
great black-backed gull/Newfoundland/296/08 (H13N2)
shorebird/Delaware/95/03 (H9N1)
duck/Hong Kong/342/78 (H5N2)
pintail/Akita/1265/08 (H1N2)
99
duck/Nanchang/1904/92 (H7N1)
duck/ Potsdam/1402-6/86 (H5N2)
mallard/Sweden/46/02 (H4N3)
shoveler/Netherlands/19/99 (H11N9)
duck/Germany/1215/73 (H2N3)
mallard/Marquenterre/Z237/83 (H1N1)
0.1
North
American
Gull
Eurasian
Wild
duck
SUPPLEMENTARY FIGURE 1
f (M)

## Slide 7
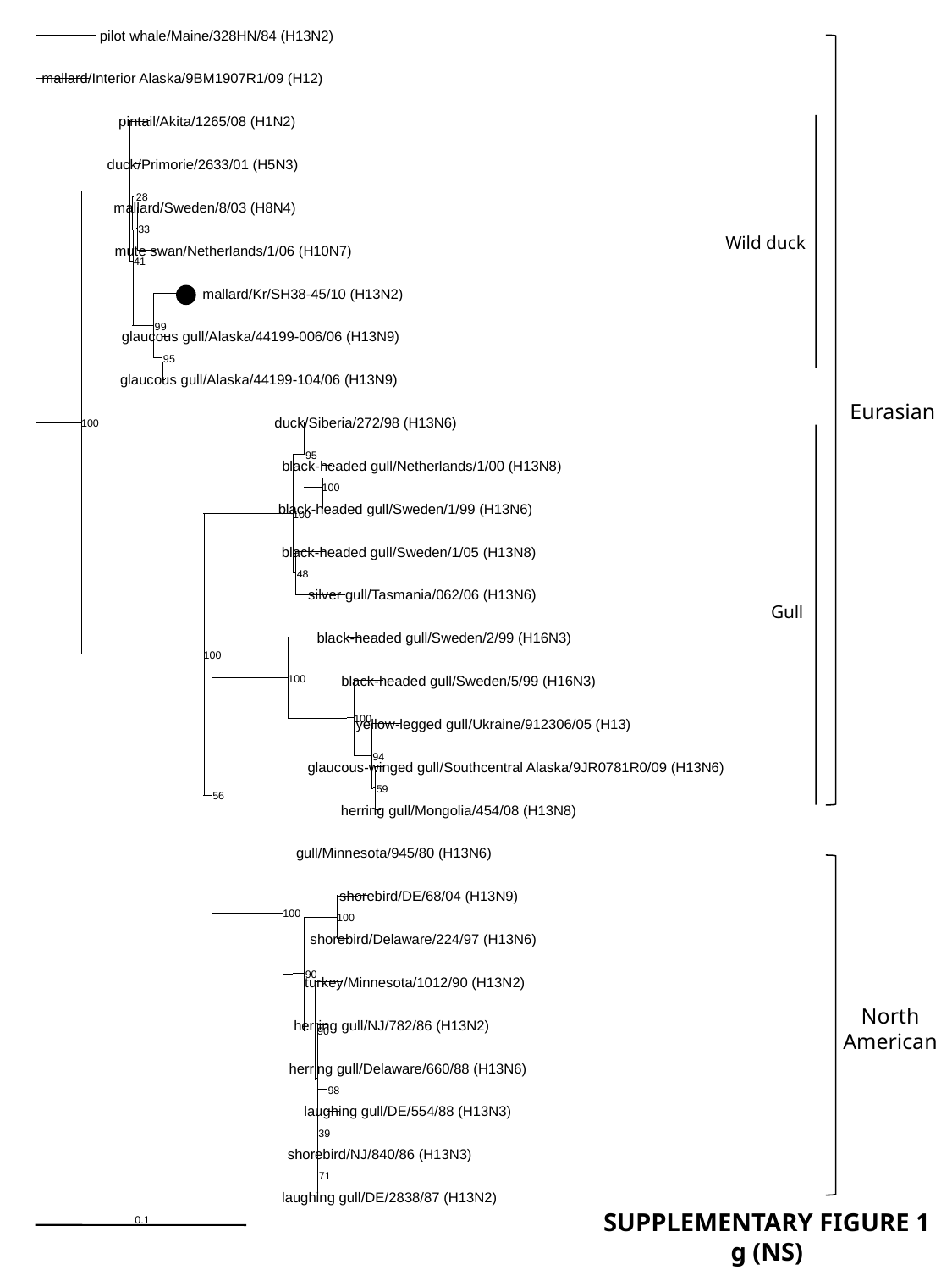

pilot whale/Maine/328HN/84 (H13N2)
mallard/Interior Alaska/9BM1907R1/09 (H12)
pintail/Akita/1265/08 (H1N2)
duck/Primorie/2633/01 (H5N3)
28
mallard/Sweden/8/03 (H8N4)
33
mute swan/Netherlands/1/06 (H10N7)
41
 mallard/Kr/SH38-45/10 (H13N2)
99
glaucous gull/Alaska/44199-006/06 (H13N9)
95
glaucous gull/Alaska/44199-104/06 (H13N9)
duck/Siberia/272/98 (H13N6)
100
95
black-headed gull/Netherlands/1/00 (H13N8)
100
black-headed gull/Sweden/1/99 (H13N6)
100
black-headed gull/Sweden/1/05 (H13N8)
48
silver gull/Tasmania/062/06 (H13N6)
black-headed gull/Sweden/2/99 (H16N3)
100
100
black-headed gull/Sweden/5/99 (H16N3)
100
yellow-legged gull/Ukraine/912306/05 (H13)
94
glaucous-winged gull/Southcentral Alaska/9JR0781R0/09 (H13N6)
59
56
herring gull/Mongolia/454/08 (H13N8)
gull/Minnesota/945/80 (H13N6)
shorebird/DE/68/04 (H13N9)
100
100
shorebird/Delaware/224/97 (H13N6)
90
turkey/Minnesota/1012/90 (H13N2)
herring gull/NJ/782/86 (H13N2)
90
herring gull/Delaware/660/88 (H13N6)
98
laughing gull/DE/554/88 (H13N3)
39
shorebird/NJ/840/86 (H13N3)
71
laughing gull/DE/2838/87 (H13N2)
0.1
Wild duck
Eurasian
Gull
North
American
SUPPLEMENTARY FIGURE 1
g (NS)
